# RepA Protein of Citrus Chlorotic Dwarf‐Associated Virus Impairs Perinuclear Chloroplast Clustering Induced by Lemon Chloroplast Malate Dehydrogenase

**DOI:** 10.1111/mpp.70133

**Published:** 2025-08-07

**Authors:** Yuan Chen, Jinfa Zhao, Jiajun Wang, Qi Zhang, Mengji Cao, Yan Zhou

**Affiliations:** ^1^ Integrative Science Center of Germplasm Creation in Western China (CHONGQING) Science City, Citrus Research Institute Southwest University/National Citrus Engineering and Technology Research Center, Citrus Research Institute, Southwest University Chongqing China

**Keywords:** chloroplast malate dehydrogenase, citrus chlorotic dwarf‐associated virus, perinuclear chloroplast clustering, replication‐related protein A

## Abstract

Replication‐related protein A (RepA), encoded by the citrus chlorotic dwarf‐associated virus (CCDaV), induces hypersensitive response (HR)‐like cell death and defence responses. However, the interactions between the host plant and CCDaV‐RepA remain unclear. In this study, *Citrus limon* chloroplast malate dehydrogenase (ClMDH) was found to interact with CCDaV‐RepA in the nucleus. ClMDH induces perinuclear chloroplast clustering (PCC). Moreover, ClMDH suppressed HR‐like cell death and the accumulation of reactive oxygen species (ROS) induced by CCDaV‐RepA, and promoted the accumulation of CCDaV‐RepA. In addition, CCDaV‐RepA overexpression altered the subcellular localisation of ClMDH from the chloroplast to the nucleus and inhibited ClMDH‐induced PCC. These results reflected the involvement of ClMDH‐induced PCC in the host response to CCDaV infection and provide new insights into the interaction between the host and CCDaV.

## Graphical abstract

RepA encoded by citrus chlorotic dwarf‐associated virus inhibits *Citrus limon* chloroplast malate dehydrogenase (ClMDH) induced perinuclear chloroplast clustering and hijacks ClMDH to the nucleus.

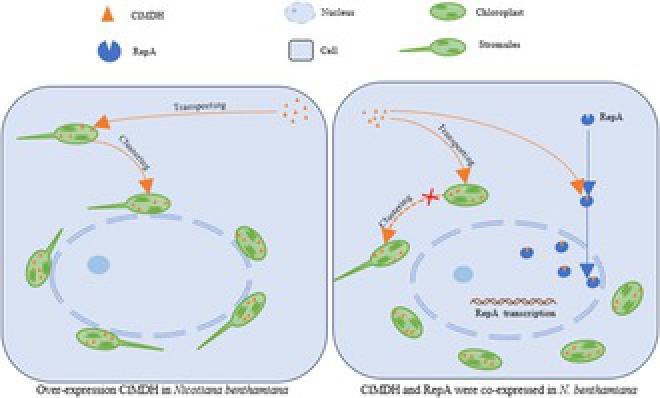

## 1 Introduction

Geminiviruses are single‐stranded (ss) circular DNA plant viruses characterised by a twin‐particle form. More than 500 species of geminiviruses have been recorded worldwide, which cause major economic losses to soybean, cassava, cotton, tomato, wheat and maize (Bonfim et al. 2007; Martin and Shepherd 2009; Patil and Fauquet 2009; Li, Qiao, et al. 2022). Recently, a new geminivirus, citrus chlorotic dwarf‐associated virus (CCDaV), was identified in citrus plants across Turkey, China, Japan and Thailand (Loconsole et al. 2012; Guo et al. 2015; Kasukabe et al. 2017). This virus is primarily transmitted through the grafting of infected buds, scions or rootstocks; it is also transmitted by *Parabemisia myricae* (Loconsole et al. 2012). CCDaV can infect most citrus genotypes; lemon ( *Citrus limon* ) and some cultivars of pummelo ( *Citrus grandis* ) are the most sensitive to this virus and develop leaf mottling, chlorosis, wrinkling and curling (Loconsole et al. 2012; Ye et al. 2024). The CCDaV genome is approximately 3.64 kb and comprises six predicted open reading frames (ORFs). However, the functions of the CCDaV proteins are poorly understood. Previous studies have shown that replication‐related protein A (RepA) encoded by its ORF4 in the antisense strand can trigger hypersensitive response (HR)‐like cell death, H_2_O_2_ burst and defence responses in *Nicotiana benthamiana* (Qin et al. 2023). The V2 protein of CCDaV inhibits single‐stranded RNA‐induced silencing (Ye et al. 2024). However, the interaction between CCDaV and its host remains unclear.

Chloroplasts are photosynthetic organelles in plants and certain algae; the structure and function of the chloroplasts may be changed by viral infection (Song et al. 2021; Bwalya and Kim 2023). For instance, infections with rice stripe virus (RSV), tomato spotted wild virus (TSWV) and barley stripe mosaic virus (BSMV) lead to chloroplast deformities and changes in reactive oxygen species (ROS), decreasing photosynthetic efficiency (Zhao et al. 2019; Wang et al. 2021; Zhan et al. 2021). In addition, tomato yellow leaf curl virus (TYLCV) infection causes severe chloroplast damage with abnormal build‐up of enlarged plastoglobuli and starch grains in tomato (Montasser et al. 2012). Similarly, radish leaf curl betasatellite (RaLCB) triggers massive plastoglobule and starch accumulation in chloroplasts of *N. benthamiana* (Bhattacharyya et al. 2015). Further study revealed that βC1 interacts with the oxygen‐evolving enhancer protein 2 (PsbP) to facilitate tomato leaf curl new delhi virus (ToLCNDV) infection (Gnanasekaran et al. 2019). Plants have evolved defensive responses to mitigate the effects of viral infections on chloroplasts (Bwalya and Kim 2023). Chloroplasts migrate to and aggregate around the nucleus under biological and abiotic stresses through the perinuclear chloroplast clustering (PCC) process (Ding et al. 2019; Zhai et al. 2021). Some studies have shown that PCC induced by the p50 of tobacco mosaic virus (TMV) and the Rep of TYLCV, beet curl top virus (BCTV) and abutilon mosaic virus (AbMV) can inhibit the movement of the virus by forming a physical barrier around the nucleus and increasing ROS accumulation, which improves plant resistance against viruses (Caplan et al. 2015; Ding et al. 2019). However, a recent study has suggested that PCC induced by the overexpression of outer membrane protein 24 (NbOMP24) promotes potato spindle tuber viroid (PSTVd) infection in *N. benthamiana* (Han, Jia, et al. 2023). Until now, the function of PCC in fruit tree virus infection is still unknown.

Malate dehydrogenase (MDH) is a highly conserved oxidoreductase commonly present in the mitochondria, cytoplasm, peroxisomes and plastids. It catalyses the interconversion between malic acid and oxaloacetic acid using NAD^+^ or NADP^+^ as cofactors, and is involved in plant growth and development, redox homeostasis and abiotic stress resistance (Moreno Garcia et al. 2022; Baird et al. 2024). MDH also plays a role in photosynthesis and disease resistance. In oak leaves, chloroplast MDH (plMDH) activity is significantly decreased, and chlorophyll synthesis is inhibited after *Oidium heveae* infection (Wang et al. 2014). Silencing *plMDH* promoted *Xanthomonas axonopodis* and *Pseudomonas syringae* pv. *tomato* DC3000‐mediated damage in cassava and *Arabidopsis thaliana* (Pant et al. 2020; Zhou et al. 2023). A previous study reported enhanced transcriptional expression of *MDH* in sugarcane infected by sugarcane mosaic virus (SCMV) (Akbar et al. 2020). Silencing *ZmMDH3* in maize can alleviate SCMV‐induced mosaic symptoms and reduce the mitochondrial ROS (mROS) levels in leaves (Jiang et al. 2023). However, the role of MDH in CCDaV infection remains unexplored.

To investigate the role of MDH in host defence mechanisms and its interaction mechanism with CCDaV RepA, the following experiments were conducted in this study. In this study, a homologous MDH from *C. limon* (ClMDH) was identified as a binding partner of CCDaV‐RepA that promotes the accumulation of CCDaV‐RepA. Moreover, CCDaV‐RepA disrupted the ability of ClMDH to induce PCC formation.

## 2 Result

### 2.1 Screening Proteins That Interact With RepA From a *Citrus limon* ‘Eureka’ cDNA Library

Considering the involvement of CCDaV RepA protein in plant defence response (Qin et al. 2023), *RepA* was cloned into the pGBKT7 vector to construct the bait vector pGBKT7‐RepA to screen potential host interaction factors from a cDNA library of *C. limon* ‘Eureka’. Autoactivation assays revealed that the co‐transformation of pGBKT7‐RepA and pGADT7 in yeast activated the *Ade* reporter gene. To suppress autoactivation, 200 ng/mL aureobasidin A (AbA) was added to the selective medium (SD/–Trp–Leu–His–Ade), and 150 candidate proteins were identified as positive in the selective medium (SD/–Tre–Leu–His–Ade+AbA). Sequence analysis revealed that chloroplast‐associated and ribosomal proteins comprised 39.34% and 18.67% of the candidate proteins, respectively; chloroplast‐associated proteins included key enzymes involved in photosynthetic pathways, such as RuBisCO, superoxide dismutase (SOD) and MDH. One of the candidate proteins, named ClMDH, exhibited 99.6% sequence similarity to the chloroplast MDH (XM 015529903.3) of *Citrus sinensis* . Considering the critical regulatory role of MDH in plant stress responses, ClMDH was further analysed.

### 2.2 Interaction Between CCDaV‐RepA and ClMDH

**FIGURE 1:**
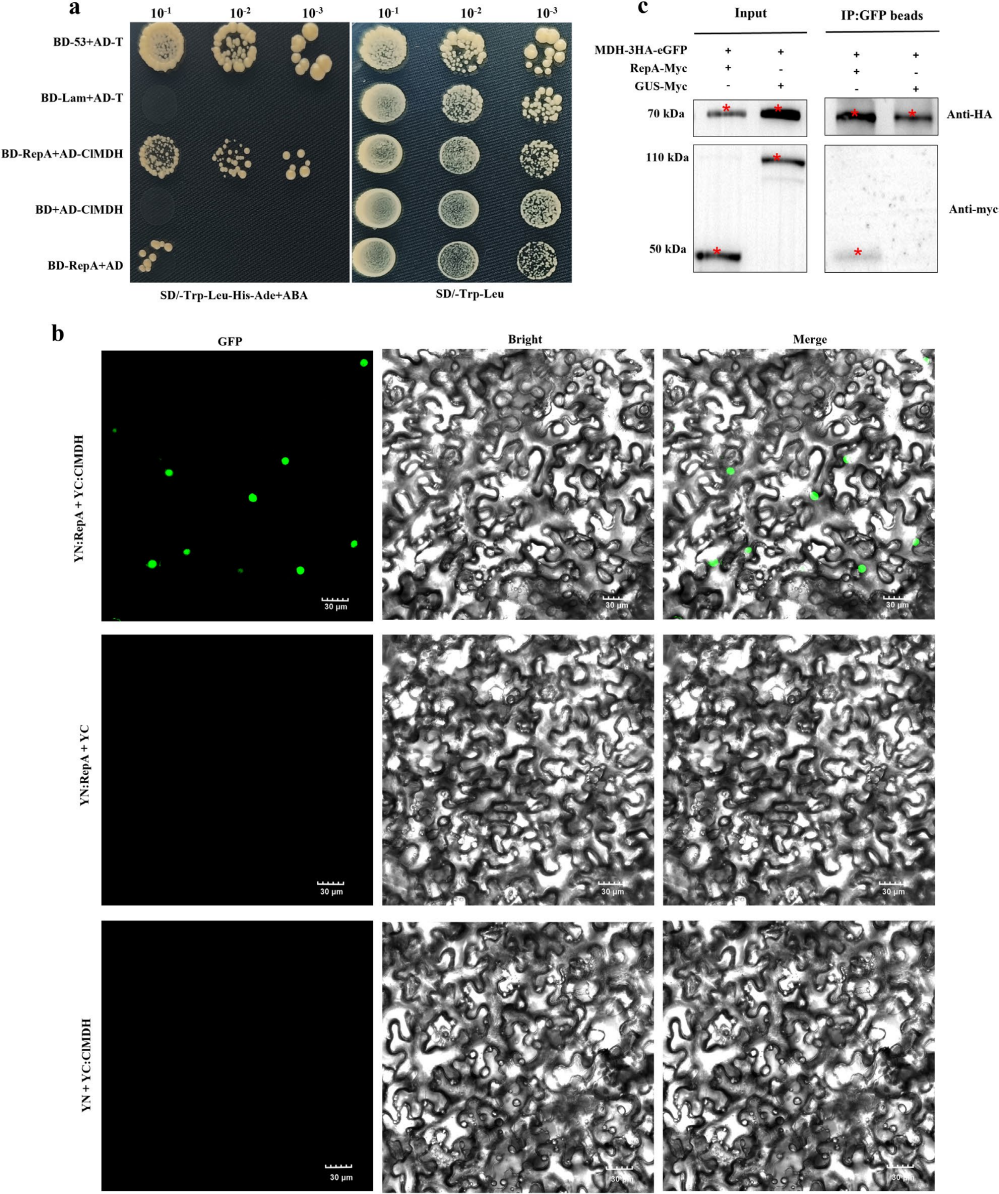
Interaction between RepA and ClMDH. (a) Yeast two‐hybrid (Y2H) analysis. BD‐RepA and AD‐ClMDH were co‐transformed into yeast competent cells. After serial dilution, the cells were transferred onto selective medium (SD/–Trp–Leu and SD/–Trp–Leu–His–Ade+AbA), followed by incubation at 28°C for 7 days. Controls included pGADT7‐ClMDH + pGBKT7 empty vector (prey plasmid auto‐activation), pGBKT7‐RepA + pGADT7 empty vector (bait plasmid auto‐activation), pGBKT7‐53 + pGADT7‐T (positive control) and pGBKT7‐Lam + pGADT7‐T (negative control). AbA, aureobasidin A. (b) Bimolecular fluorescence complementation (BiFC) analysis. RepA was fused to the N‐terminal of plasmid pCV‐YFP, while ClMDH was fused to the C‐terminal. The plasmids were transformed into *Agrobacterium tumefaciens* GV3101 and co‐expressed in *Nicotiana benthamiana* via *Agrobacterium* infiltration. Fluorescence was detected at 48 h post‐infiltration (hpi). Negative controls included YN‐RepA + YC and YN + YC‐ClMDH. Scale bar, 30 μm. (c) Co‐immunoprecipitation (Co‐IP) analysis. Plasmids pART27‐RepA‐myc, pART27‐GUS‐myc and pART27‐ClMDH‐3HA‐eGFP were transformed into the GV3101 strain. pART27‐ClMDH‐3HA‐eGFP was co‐expressed with pART27‐RepA‐myc or pART27‐GUS‐myc in *N. benthamiana* via *Agrobacterium* infiltration; samples were collected at 48 hpi. Crude extracts were immunoprecipitated using anti‐GFP beads, and immunoblotting was performed with anti‐HA and anti‐myc antibodies for input and Co‐IP samples. Each experiment was repeated three times, and three biological replicates were analysed for each repetition.

To validate the interaction between RepA and ClMDH in yeast, the full‐length coding sequence (CDS) of *ClMDH* was cloned into the pGADT7 vector to construct a pGADT7‐ClMDH prey plasmid. Next, this prey plasmid was co‐transformed with pGBKT7‐RepA into competent yeast cells for yeast two‐hybrid (Y2H) analysis. As expected, yeast colonies co‐transformed with pGBKT7‐RepA and pGADT7‐ClMDH grew on the selective medium (SD/–Trp–Leu–His–Ade+AbA), whereas those co‐transformed with pGADT7‐ClMDH and pGBKT7 failed to grow (Figure 1a). These results confirmed that RepA and ClMDH interact in yeast. Subsequently, bimolecular fluorescence complementation (BiFC) analysis allowed further verification of interactions between CCDaV‐RepA and ClMDH. pCV‐nYFP‐RepA and pCV‐cYFP‐ClMDH were constructed and co‐expressed via *Agrobacterium* infiltration into *N. benthamiana* leaves. Confocal microscopy revealed green fluorescence in the nucleus at 48 h post‐infiltration (hpi). No fluorescence was observed in the negative controls co‐expressing pCV‐nYFP‐RepA with pCV‐cYFP or pCV‐nYFP with pCV‐cYFP‐ClMDH (Figure 1b). For co‐immunoprecipitation (Co‐IP) assays, pART27‐ClMDH‐3HA‐eGFP was co‐expressed with either pART27‐RepA‐myc or pART27‐GUS‐myc in *N. benthamiana*. Protein extracts were precipitated using Anti‐eGFP Magnetic Beads, followed by SDS‐PAGE and immunoblotting. The results showed that RepA‐myc coprecipitated with ClMDH‐3HA‐eGFP; no coprecipitation was detected in the negative control (co‐expressing GUS‐myc and ClMDH‐3HA‐eGFP) (Figure 1c). These findings confirmed the in vivo interaction between RepA and ClMDH.

### 2.3 Identification of the Key Region Involved in ClMDH–RepA Interaction

**FIGURE 2:**
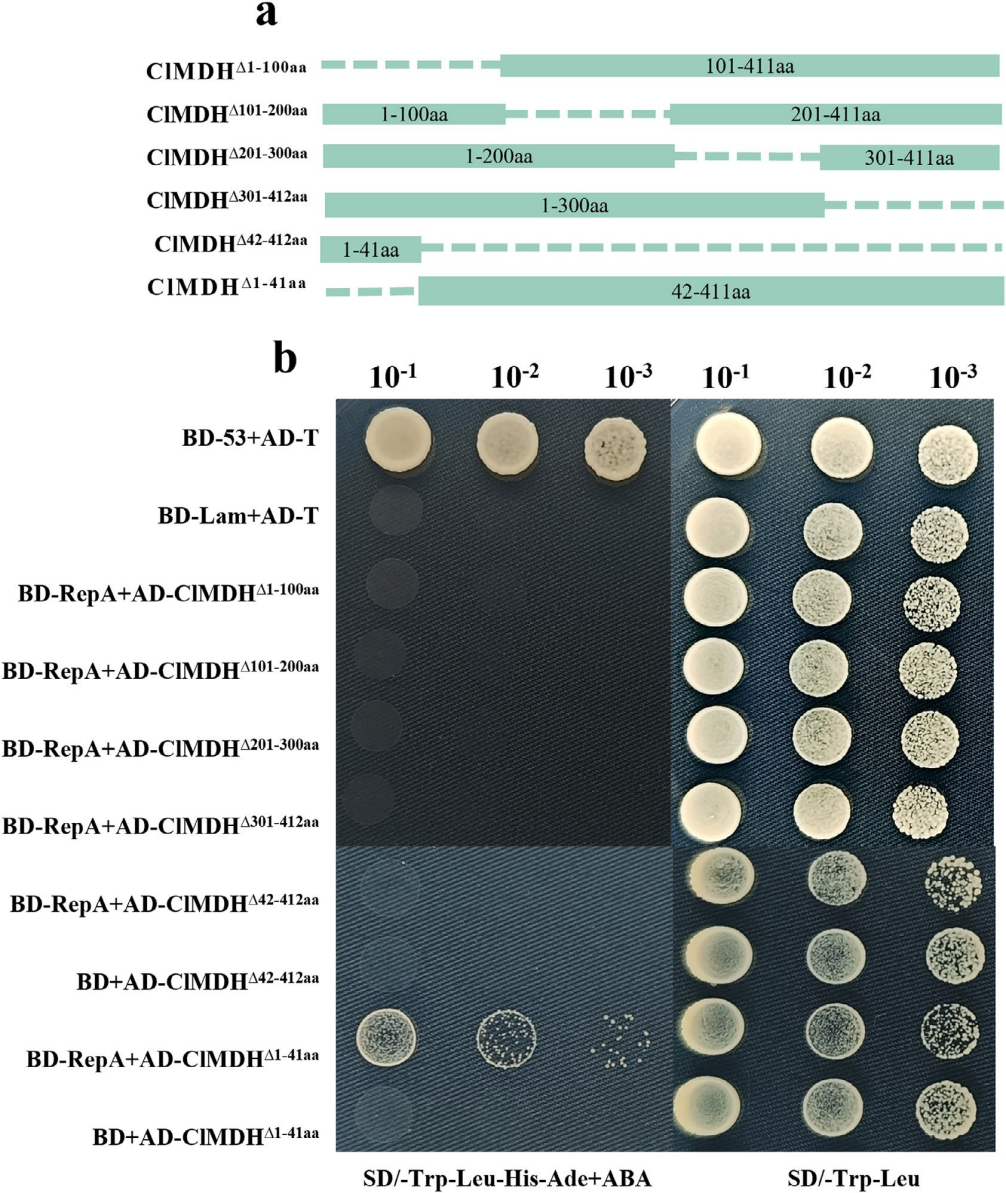
Identification of the critical region involved in the interaction between ClMDH and RepA. (a) Schematic diagram of truncated ClMDH constructs. (b) Yeast two‐hybrid (Y2H) analysis of interactions between truncated ClMDH mutants and RepA. pGBKT7‐53 + pGADT7‐T and pGBKT7‐Lam + pGADT7‐T served as the positive and negative controls, respectively. Each experiment included three repeats, and three biological replicates were included in each repetition.

To identify the key regions of ClMDH that regulate the interactions with RepA, four truncated ClMDH mutants (ClMDH^∆1–100aa^, ClMDH^∆101‐200aa^, ClMDH^∆201‐300aa^ and ClMDH^∆300‐412aa^) were co‐transformed with pGBKT7‐RepA into Y2H Gold yeast. However, none of the ClMDH mutants interacted with RepA on the selective medium (SD/–Leu–Trp–His–Ade+AbA) (Figure 2). Previous studies suggested that the N‐terminus of the chloroplast protein contains a 30–80 amino acid (aa) transit peptide that regulates its import into chloroplasts (Zhang and Glaser 2002; Liu et al. 2024) and that the chloroplast transit peptide (cTP) is not essential for the interaction of chloroplast proteins with viral proteins (Cheng et al. 2021). Therefore, we disrupted the cTP of ClMDH and generated two truncated mutants: ClMDH^∆1‐41aa^ and ClMDH^∆42‐412aa^. Subcellular localisation results revealed that ClMDH^∆1‐41aa^ and ClMDH^∆42‐412aa^ could not localise to chloroplasts (Figure S1). Y2H assays revealed that yeast co‐transformed with pGADT7‐ClMDH^∆1‐41aa^ and pGBKT7‐RepA grew on the selective medium (SD/–Trp–Leu–His–Ade+AbA), while yeast co‐transformed with pGADT7‐ClMDH^∆42‐412aa^ and pGBKT7‐RepA or pGBKT7 with the truncated ClMDH mutants, failed to grow (Figure 2). This suggests that the region of 42–412 aa is crucially involved in the interaction between RepA and ClMDH.

### 2.4 Homology Analysis of the ClMDH Family and cis‐Regulatory Element Analysis

Based on data available in the online platform CPBD (http://citrus.hzau.edu.cn/), we retrieved the protein sequences homologous to ClMDH, derived from five *Citrus* varieties: *C. australasica* (Egl075920), *C. grandis* (Cg5g010240), *C. reticulata* (MSYJ175910), *C. reticulata* (Cme164120) and *C. clementina* (Ciclev10020378m). The MDH family protein sequences of *C. sinensis* , *Zea mays* , *Oryza sativa* and *A. thaliana* were obtained from the National Center for Biotechnology Information (NCBI) website (https://www.ncbi.nlm.nih.gov/). The obtained protein sequences were compared using multiple sequence alignments, and an unrooted phylogenetic tree was constructed. The analysis revealed that ClMDH is associated with the plNAD‐MDH isoform of the MDH subfamily II (Figure S2a). The sequence of ClMDH exhibited 98.06%–100% similarity with *Citrus* plNAD‐MDH and 53.11%–86.23% similarity with plNAD‐MDH from *Z. mays* , *O. sativa* and *A. thaliana* (Figure S3).

Additionally, we submitted the promoter sequences of MDHs from *C. sinensis* to the PlantCARE online platform (https://bioinformatics.psb.ugent.be/webtools/plantcare/html/) for cis‐regulatory element (CRE) prediction. The plNAD‐MDH promoter contains drought‐responsive, low‐temperature‐responsive and TC‐rich repeats (Figure S2b). These results imply that ClMDH, a plNAD‐MDH isoform, is involved in plant responses to biotic and abiotic stressors.

### 2.5 Expression Pattern of Homologous *MDH* in *Citrus* Species

**FIGURE 3:**
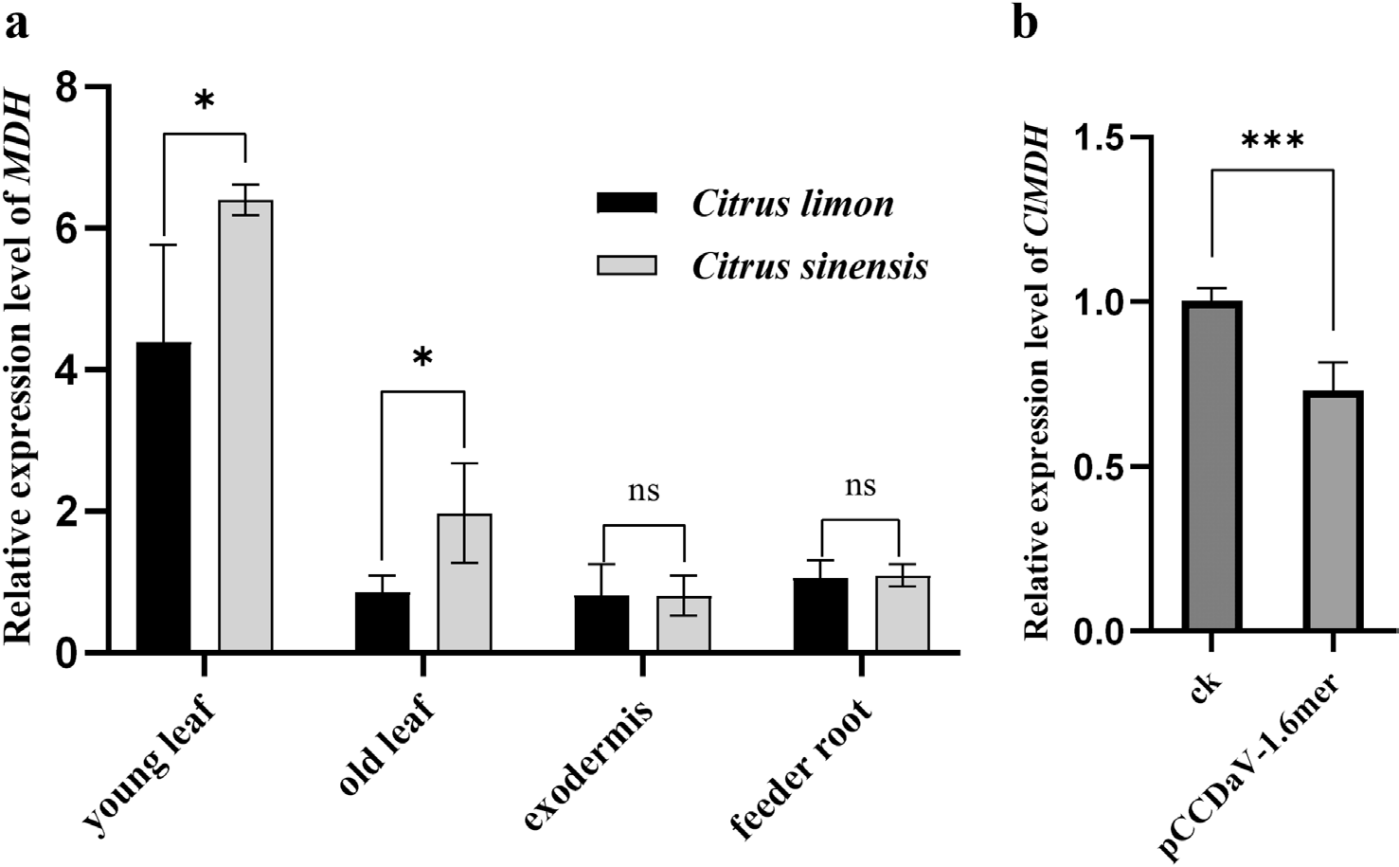
Expression profile of homologous *MDH* in *Citrus* spp. (a) Expression levels of *MDH* in resistant ( *C. sinensis* ) and susceptible ( *C. limon* ) varieties. (b) CCDaV‐infection downregulated *ClMDH* expression. CK: Virus‐free lemon. Reverse transcription‐quantitative PCR analysis was performed at 8 days post‐infection (dpi). Student's *t* test was used to determine statistically significant differences between the two samples (*p < 0.05, ***p < 0.001). Each experiment was repeated three times, and each repeat included three biological replicates.

To investigate the expression profile of homologous *MDH* in different *Citrus* species, total RNA was extracted from young leaves, old leaves, exodermis and feeder roots of 1‐year‐old virus‐free lemon (sensitive to CCDaV) and virus‐free sweet oranges (resistant to CCDaV). Reverse transcription‐quantitative PCR (RT‐qPCR) analysis revealed that the expression of homologous *MDH* was significantly higher in the young leaves of lemon and sweet orange than in the old leaves, exodermis and feeder roots (Figure 3a). Furthermore, the expression level of homologous *MDH* was significantly higher in young and old leaves of sweet orange than in the counterparts acquired from lemons; however, the relative expression levels in the exodermis and feeder roots did not significantly differ between sweet orange and lemon (Figure 3a).

To investigate the effects of CCDaV infection on the expression level of *ClMDH*, lemon leaves were inoculated with an infectious clone of CCDaV (pCCDaV‐1.6 mer) via *Agrobacterium* infiltration. The RT‐qPCR analysis of inoculated tissues revealed that *ClMDH* mRNA levels in CCDaV‐infected lemons decreased by 26.61% compared to the control counterparts (Figure 3b).

### 2.6 ClMDH Localises to the Chloroplast and Induces PCC

**FIGURE 4:**
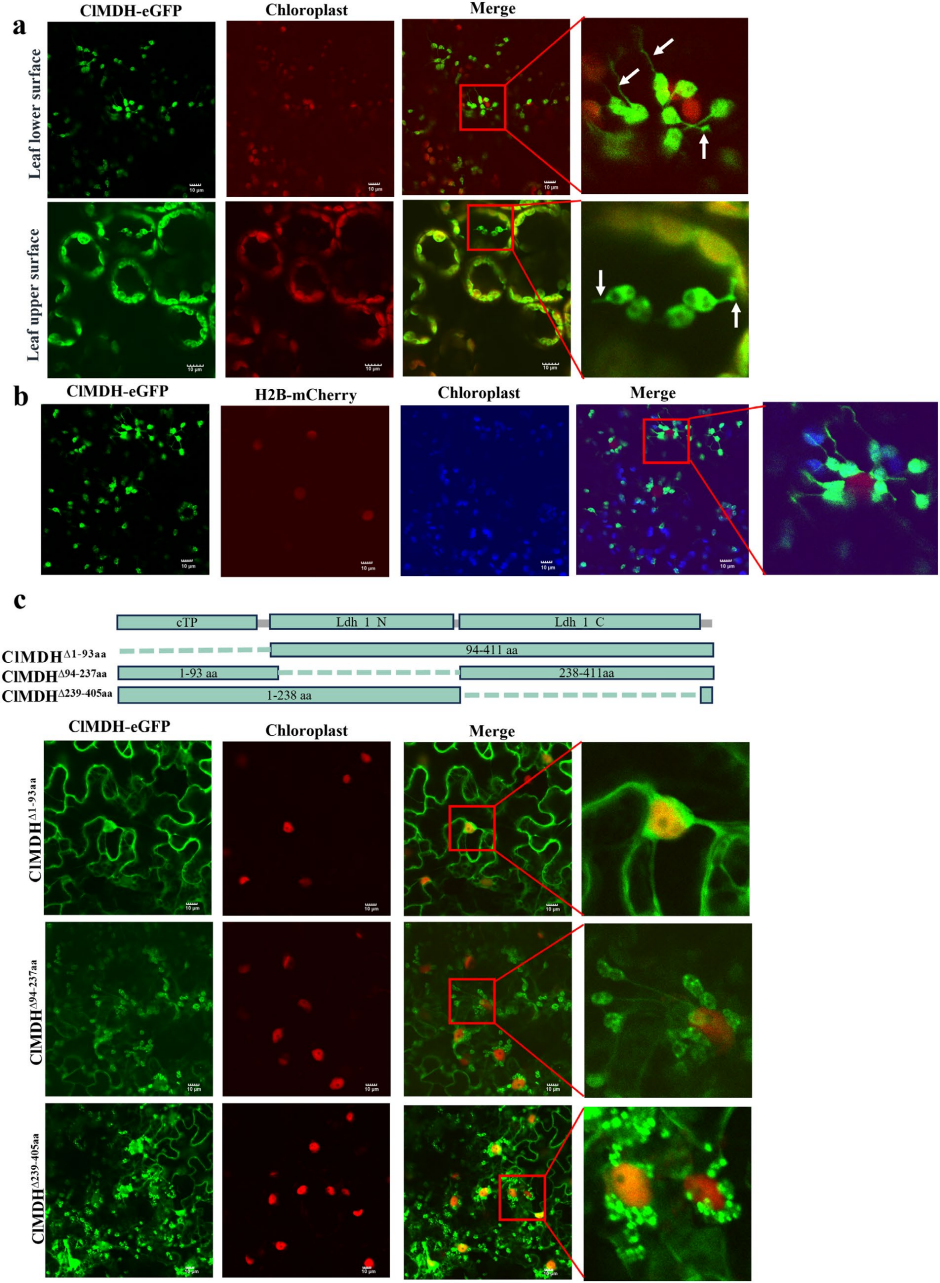
ClMDH localises to chloroplasts and induces perinuclear chloroplast clustering (PCC). (a) ClMDH is localised in chloroplasts. The pATR27‐ClMDH‐eGFP was transiently expressed in *Nicotiana benthamiana* via *Agrobacterium* infiltration, and fluorescence was observed at 40 h post‐infiltration (hpi). Chloroplast autofluorescence is indicated in red. White arrows indicate the stromules. Scale bar, 10 μm. (b) ClMDH induces PCC. ClMDH‐eGFP and H2B‐mCherry (a nuclear marker with red fluorescence) were co‐expressed in *N. benthamiana* via *Agrobacterium* infiltration. Chloroplast aggregation around nuclei was visualised using confocal microscopy at 40 hpi; blue indicates chloroplast autofluorescence. Scale bar, 10 μm. (c) Subcellular localisation of ClMDH truncation mutants. The truncated ClMDH mutants fused with eGFP were co‐expressed with H2B‐mCherry in *N. benthamiana*. Fluorescence was observed at 40 hpi. Scale bar, 10 μm. Each experiment was repeated three times, and each repeat included three biological replicates.

We predicted the subcellular localisation of ClMDH using the online platform WoLF PSORT II (https://www.genscript.com/wolf‐psort.html), which predicted its localisation to the chloroplast. Subsequently, we fused ClMDH to the N‐terminus of eGFP in the binary vector pART27‐eGFP to generate pART27‐ClMDH‐eGFP, and transiently expressed it in *N. benthamiana* via *Agrobacterium* infiltration. Confocal microscopic analysis at 40 h post‐infiltration (hpi) revealed that the green fluorescence of ClMDH‐eGFP overlapped with the red autofluorescence corresponding to chloroplasts, which confirmed that ClMDH was localised in the chloroplast (Figure 4a). Notably, the chloroplasts labelled with green fluorescence exhibited extended stromules (Figure 4a).

In *N. benthamiana*, the overexpression of chloroplast‐localised proteins induces chloroplast‐derived stromules and PCC, facilitating ROS signal transmission from the chloroplast to the nucleus, and activating plant defence responses (Caplan et al. 2015; Cheng et al. 2021; Zhai et al. 2021). To investigate whether ClMDH overexpression induces PCC, ClMDH‐eGFP was co‐expressed with the nuclear marker H2B‐mCherry in *N. benthamiana*. ClMDH‐eGFP‐labelled chloroplasts aggregated around the nuclei and formed stromules at 40 hpi, indicating the induction of PCC (Figure 4b). We identified the functional domain of ClMDH using SMART (https://smart.embl.de/) to predict the presence of the Ldh_1_N (94–237 aa) and Ldh_1_C (239–405 aa) domains (Figure 4c). To validate the domain of ClMDH, which is involved in inducing PCC, three truncated mutants (ClMDH^∆1‐93aa^, ClMDH^∆94‐237aa^ and ClMDH^∆239‐405aa^) fused with eGFP were separately co‐expressed with H2B‐mCherry in *N. benthamiana*. ClMDH^∆1‐93aa^ was localised to the nucleus and cytoplasm; however, other truncated mutants were detected in the chloroplast and induced chloroplast clustering around the nucleus. Additionally, ClMDH^∆239‐405aa^ could not induce chloroplast stromules (Figure 4c). These results indicated the crucial role of residues 1–93 aa in chloroplast targeting and PCC induction; moreover, the Ldh_1_C domain (239–405 aa) participates in stromule formation.

### 2.7 ClMDH Attenuated RepA‐Induced ROS Accumulation and HR‐Like Necrosis

**FIGURE 5:**
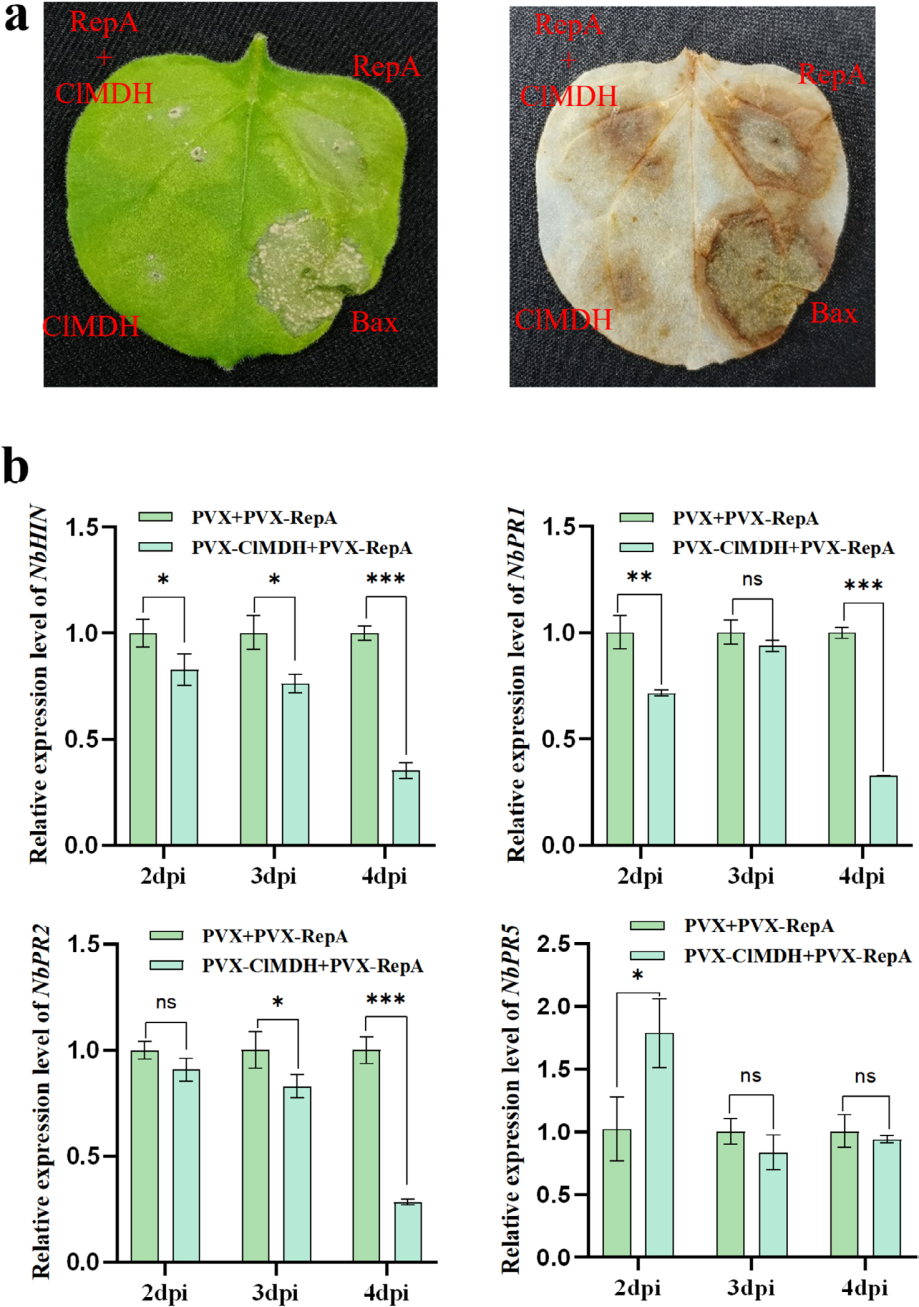
ClMDH attenuated RepA‐induced reactive oxygen species (ROS) accumulation and necrosis. (a) Effect of ClMDH on RepA‐induced hypersensitive response (HR)‐like necrosis. Leaves were analysed using 3,3′‐diaminobenzidine (DAB) staining at 7 days post‐inoculation (dpi). (b) Changes in defence‐related gene expression. Student's *t* test was used to determine significant differences (*p < 0.05, **p < 0.01, ***p < 0.001). Each experiment was repeated three times, and each repeat included three biological replicates.

PCC is consistently accompanied by the accumulation of ROS (Caplan et al. 2015; Ding et al. 2019; Zhai et al. 2021), and CCDaV‐RepA can induce ROS bursts and HR‐like necrosis in *N. benthamiana* (Qin et al. 2023). To investigate whether *ClMDH* overexpression induces ROS accumulation and affects RepA‐triggered HR‐like necrosis, PVX‐RepA‐myc, PVX‐ClMDH‐HA and PVX‐Bax were infiltrated in *N. benthamiana* leaves through *Agrobacterium*. At 7 days post‐inoculation (dpi), 3,3′‐diaminobenzidine (DAB) staining analysis of infiltrated leaves revealed that RepA expression induced ROS accumulation and necrosis. *ClMDH* expression promoted ROS accumulation but did not cause necrosis (Figure 5a). The co‐expression of *ClMDH* with *RepA* attenuated RepA‐induced ROS accumulation and necrosis compared to those detected after the expression of RepA alone (Figure 5a). ROS act as signalling molecules in salicylic acid (SA)‐mediated defence responses (Jiang et al. 2022; Xu et al. 2024). RT‐qPCR analysis of the infiltrated leaves revealed that the expression levels of the pathogenesis‐related gene *NbHIN* and the SA pathway‐associated defence genes *NbPR1* and *NbPR2* were significantly downregulated compared with the negative control counterparts at 4 dpi (Figure 5b).

### 2.8 ClMDH Positively Regulates RepA Transcription and Promotes RepA Accumulation

**FIGURE 6:**
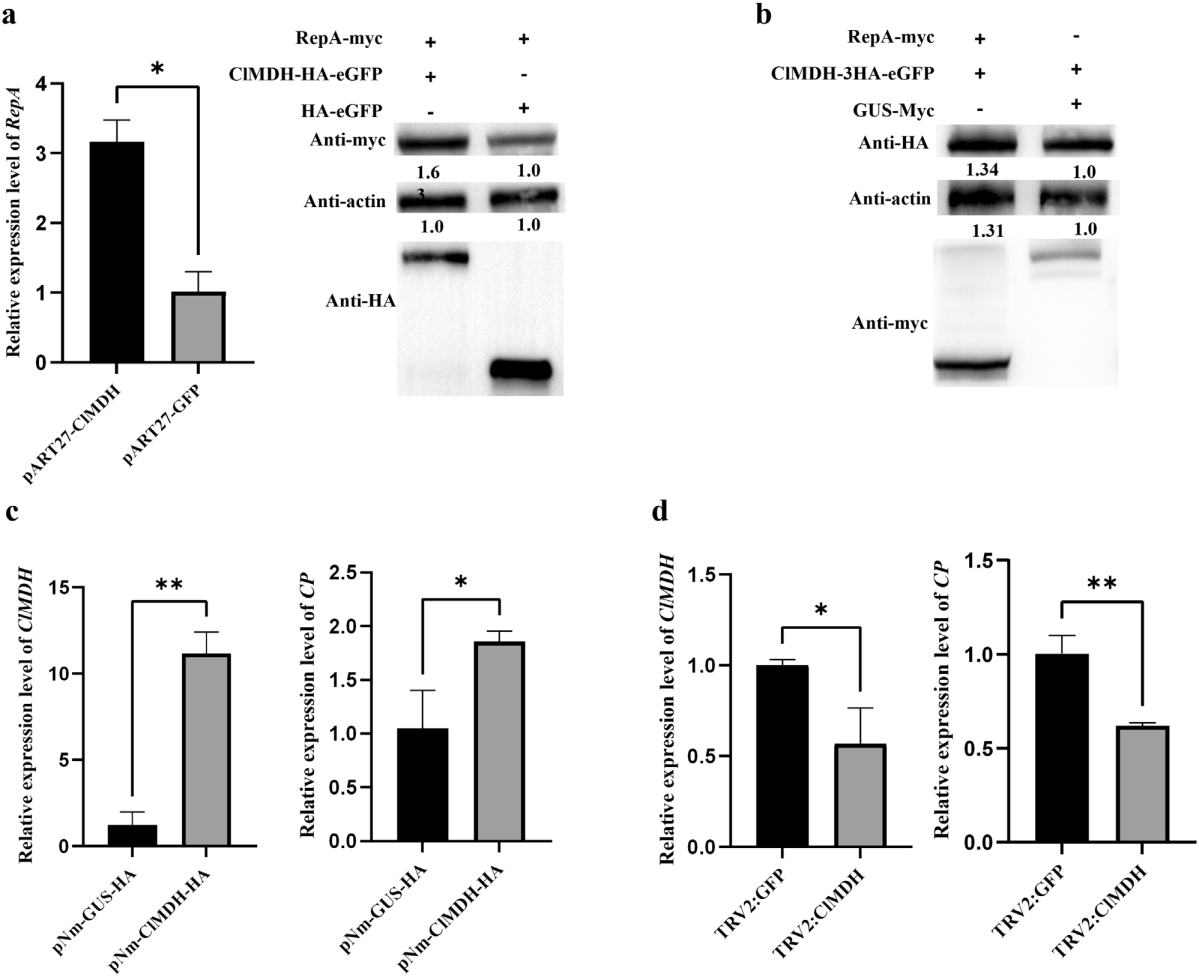
ClMDH positively regulates *RepA* transcription and resistance to CCDaV. (a) Transient overexpression of *ClMDH* promotes *RepA* transcription and RepA accumulation in *Nicotiana benthamiana*. RepA‐myc and ClMDH‐3HA‐eGFP were co‐expressed in *N. benthamiana* via *Agrobacterium* infiltration; 3HA‐eGFP was considered a control. Total RNA and protein were extracted at 48 h post‐infiltration (hpi). Reverse transcription‐quantitative PCR (RT‐qPCR) and western blot analyses were conducted. Significant differences were determined using Student's *t* test (**p < 0.01). (b) RepA did not affect the ClMDH protein accumulation level. We co‐expressed pART27‐RepA‐myc and pART27‐ClMDH‐HA‐eGFP in *N. benthamiana* via *Agrobacterium* infiltration, considering the co‐expression of pART27‐GUS‐myc and pART27‐ClMDH‐HA‐eGFP as the control. (c) Transient overexpression of *ClMDH* enhances *CP* transcription in *Citrus limon* . pNmGFPer‐ClMDH‐HA was expressed in lemon leaves via *Agrobacterium* infiltration. At 5 days post‐infiltration (dpi), leaves were inoculated with pCCDaV‐1.6mer; 2 days after inoculation, samples were collected for RT‐qPCR analysis. (d) Silencing *ClMDH* suppresses *CP* mRNA levels in *C. limon* . The TRV vector was used to silence *ClMDH* in lemon. At 60 dpi, pCCDaV‐1.6mer was inoculated into *ClMDH*‐silenced and control plants; 5 days after inoculation, RNA was extracted for RT‐qPCR. Significant differences were determined using Student's *t* test (*p < 0.05, **p < 0.01). Each experiment was repeated three times, and each repeat included three biological replicates.

Viruses hijack host proteins to facilitate replication (Hyodo and Okuno 2020; Cheng et al. 2021; Rodriguez‐Pena et al. 2021). To investigate the effect of ClMDH on RepA, pART27‐RepA‐myc + pATR27‐ClMDH‐HA‐eGFP and pART27‐RepA‐myc + pART27‐HA‐eGFP were transformed into the GV3101 strain, which was infiltrated into *N. benthamiana*. RT‐qPCR and western blot analyses showed that *RepA* mRNA level and content were upregulated by ClMDH at 48 hpi (Figure 6a). Additionally, pART27‐ClMDH‐HA‐eGFP was co‐expressed in *N. benthamiana* leaves with either pART27‐RepA‐myc or pART27‐GUS‐myc. Western blot analysis of samples collected at 48 hpi revealed that RepA did not affect ClMDH protein levels (Figure 6b).

### 2.9 ClMDH Negatively Regulates Resistance to CCDaV

To determine the effect of ClMDH on *Citrus* plant resistance to CCDaV infection, ClMDH was transiently expressed in *C. limon* using the pNmGFPer‐3HA vector. The expression level of *ClMDH* increased at 5 dpi by approximately 9.10‐fold compared to the control counterparts (Figure 6c). Subsequently, the infiltrated leaves were inoculated using the CCDaV infectious clone (pCCDaV‐1.6mer); 2 days after inoculation, the transcriptional level of *CP* was 1.77 times higher than that in the control (Figure 6c). Additionally, *ClMDH* was silenced in *C. limon* using a TRV vector as previously described. RT‐qPCR revealed that the *ClMDH* expression level was reduced by 50.23% at 60 dpi compared to the control counterpart (Figure 6d). *ClMDH*‐silenced leaves were inoculated using the infectious CCDaV clone; 5 days after inoculation, the transcriptional level of *CP* was reduced by 37.84% compared to the control counterpart (Figure 6d).

### 2.10 RepA Impairs ClMDH‐Induced PCC and Relocalises ClMDH to the Nucleus

**FIGURE 7:**
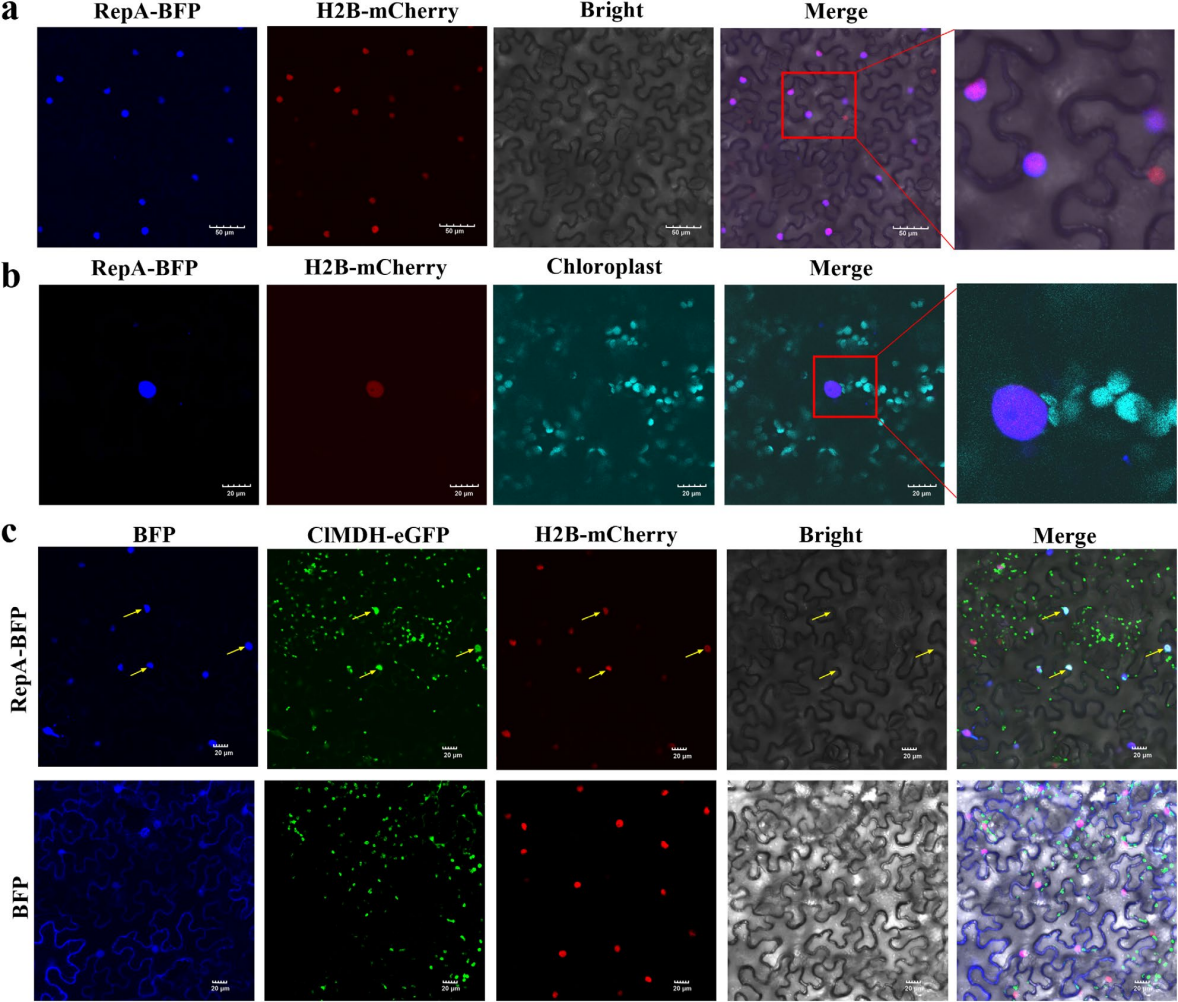
Subcellular localisation of RepA and its co‐localisation with ClMDH. (a) RepA is localised in the nucleus. Scale bars, 50 μm. (b) RepA cannot induce perinuclear chloroplast clustering (PCC). H2B‐mCherry is a nuclear marker with red fluorescence. Cyan fluorescence is chloroplast autofluorescence. Scale bars, 20 μm. (c) Co‐localisation of RepA and ClMDH in the nucleus. H2B‐mCherry is a nuclear marker with red fluorescence. Scale bar, 20 μm. Each experiment was repeated three times, and each repeat included three biological replicates.

**FIGURE 8:**
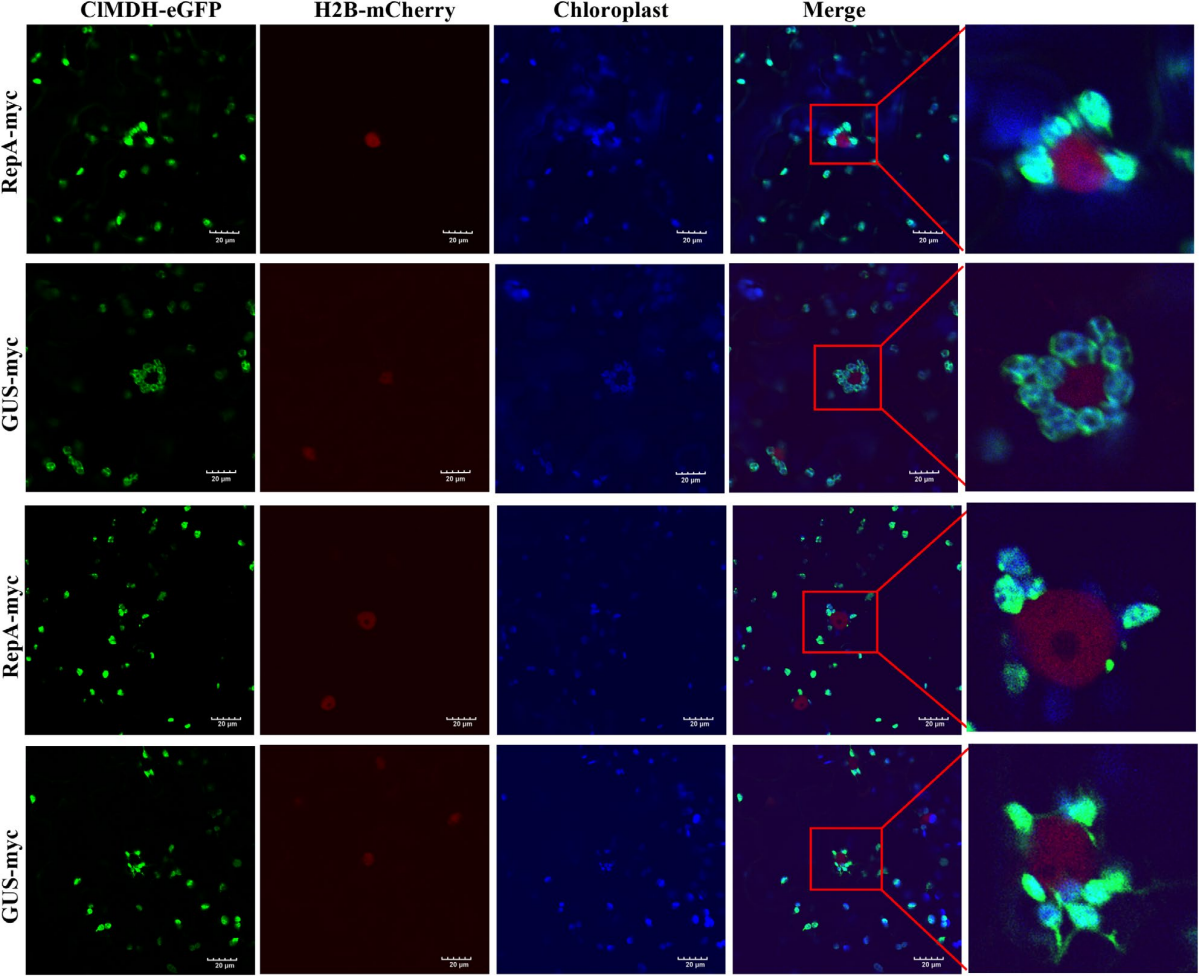
RepA impairs ClMDH‐induced perinuclear chloroplast clustering (PCC) . PCC was defined as > 3 chloroplasts surrounding the nucleus. RepA‐myc and ClMDH‐eGFP were co‐expressed in *N. benthamiana* via *Agrobacterium* infiltration, considering GUS‐myc a control. Fluorescence and chloroplast counts were analysed at 40 hpi. H2B‐mCherry is a nuclear marker with red fluorescence. Blue fluorescence is chloroplast autofluorescence. Scale bar, 20 μm. Each experiment was repeated three times, and each repeat included three biological replicates.

To further investigate whether RepA disrupts the ClMDH‐induced PCC, pCV‐RepA‐BFP was co‐expressed with H2B‐mCherry in *N. benthamiana*, which revealed that RepA was localised to the nucleus and failed to induce PCC (Figure 7a,b). Subsequently, the cells were co‐infiltrated with pART27‐RepA‐myc and pART27‐ClMDH‐HA‐eGFP via *Agrobacterium* infiltration. Compared with the control (pART27‐GUS‐myc + pART27‐ClMDH‐HA‐eGFP), pART27‐RepA‐myc + pART27‐ClMDH‐HA‐eGFP markedly suppressed the PCC triggered by ClMDH and inhibited the ability of ClMDH to promote stromules formation at 48 hpi (Figure 8). These results suggested that RepA impaired ClMDH‐induced PCC. Simultaneously, western blotting revealed the expression levels of the target proteins (Figure S4). MDH typically assembles to form a homodimer associated with its enzymatic activity (Baird et al. 2024). To demonstrate whether RepA attenuates ClMDH‐mediated PCC through the disrupted homodimer assembly, we tested the ability of ClMDH to form homodimers. ClMDH did not self‐interact in the Y2H assay. The chloroplast localisation of MDH is crucial for its ability to induce PCC. We explored whether RepA recruits ClMDH to the nucleus through the co‐expression of pCV‐RepA‐BFP, pART27‐ClMDH‐eGFP and H2B‐mCherry. The green fluorescence indicating ClMDH‐eGFP shifted to the nucleus at 48 hpi (Figure 7c), indicating that RepA redirected chloroplast‐localised ClMDH to the nucleus.

## 3 Discussion

CCDaV is a newly discovered geminivirus first identified in 2012 through deep sequencing (Loconsole et al. 2012). CCDaV is considered the most dangerous viral pathogen of citrus plants in Turkey, causing heavy losses to the grapefruit industry (Loconsole et al. 2012). Recently, it has been reported to significantly affect the production of pummelos and lemons in China (Guo et al. 2015; Ye et al. 2024).

Understanding virus–host interactions can provide an important theoretical basis for revealing the pathogenesis of viruses and help improve host resistance. However, the pathogenic mechanism of CCDaV in *Citrus* cells remains unclear. In the present study, we identified ClMDH as a RepA‐binding partner in the cDNA library of Eureka lemon. Additionally, the expression level of *ClMDH* was downregulated after CCDaV infection. These results indicate that ClMDH participates in plant responses to CCDaV infection. Previously, MDH was reported to participate in plant growth and development (Selinski et al. 2014; Wu et al. 2019; Chen et al. 2020; Nan et al. 2020; Yokochi et al. 2021; Tian et al. 2022; Shi, Feng, et al. 2023; Baird et al. 2024), and enhance plant resistance to pathogens, such as *P. syringae* pv. *tabaci*, *X. axonopodis* pv. *manihotis* and SCMV, by promoting malic acid accumulation (Pant et al. 2020; Jiang et al. 2023; Zhou et al. 2023). In the present study, we confirmed ClMDH‐induced PCC in plants. Moreover, 1–93 aa has been identified as a critical region for chloroplast targeting and PCC induction, and the Ldh_1_C domain is essential for stromule formation. These results contribute to the comprehensive knowledge of ClMDH functions.

In the process of co‐evolution with viruses, plants have developed a variety of defence mechanisms, among which the PCC is crucially associated with an antiviral response. Viruses have evolved strategies to successfully infect plants by overcoming plant resistance mediated by chloroplast aggregation. For instance, the VPg protein of the turnip mosaic virus (TuMV) promotes viral infection by impairing the PCC induced by the chloroplast NADH dehydrogenase‐like complex M subunit (Zhai et al. 2021). Similarly, the coat protein (CP) of the pepper mild mottle virus (PMMoV) disrupts PCC mediated by the chloroplast outer envelope membrane protein (NbOMP24) in *N. benthamiana*, facilitating PMMoV proliferation (Han, Zheng, et al. 2023). Han, Jia, et al. (2023) demonstrated that overexpression of *NbOMP24* induced PCC in *N. benthamiana* and significantly increased PSTVd content in plants. These results indicate a complex regulatory system of plant resistance to viruses through PCC. In this study, we elucidated that ClMDH induced PCC and ClMDH negatively regulated lemon resistance to CCDaV. In the future, ClMDH mutants that do not induce PCC will be generated in lemons to further investigate the role of PCC in plant responses to CCDaV infection. The expression levels of homologous *MDH* in leaves were higher in CCDaV‐resistant sweet orange than in CCDaV‐sensitive lemons, which indicates that multiple resistance pathways are potentially involved in the plant response to CCDaV infection.

ROS play a significant role in defence reactions against plant viruses. Liao et al. (2024) reported that ROS bursts can inhibit the citrus yellow vein clearing virus (CYVCV) in lemon (Liao et al. 2024). The ClAPX1‐mediated decomposition of H_2_O_2_ increases the CYVCV accumulation in plants (Wang et al. 2023). Plant viruses can also utilise ROS to promote infection or replication. For instance, the replication of red clover necrotic mosaic virus (RCNMV) and brome mosaic virus (BMV) depends on the involvement of ROS derived from respiratory burst oxidase homologues (RBOH) (Hyodo et al. 2017). In the present study, the accumulation of H_2_O_2_ was reduced in leaves co‐expressing RepA and ClMDH, and HR‐like cell death was alleviated compared to that in leaves that overexpressed RepA; the accumulation of RepA was promoted by the expression of ClMDH. These results indicate that the ROS‐mediated defence response may negatively regulate the accumulation of RepA and CCDaV infection in plants.

In this study, we detected that amino acid residues 1–93 at the N‐terminus of ClMDH were critical for chloroplast targeting and PCC induction; moreover, the Ldh_1_C domain (239–405 aa) was essential for stromule formation. Additionally, Ldh_1_N and Ldh_1_C (42–412aa) were involved in the interaction between ClMDH and RepA. These results indicate that the ClMDH–RepA interaction potentially affects the ability of ClMDH to induce a PCC response. We plan to further explore this mechanism.

In conclusion, these results reflect that ClMDH interacts with CCDaV RepA in the nucleus. CCDaV RepA impairs ClMDH‐induced PCC function and hijacks ClMDH from the chloroplast to the nucleus. Because ClMDH can promote the accumulation of CCDaV, it provides a potential target for obtaining disease‐resistant plants through gene‐editing technology. This study provides novel insights into the role of ClMDH in mediating plant defence responses against CCDaV infections.

## 4 Experimental Procedures

### 4.1 Plant Materials and Growth Conditions

*Nicotiana benthamiana* and seedlings of *C. limon* ‘Eureka’ were cultivated at 25°C in a growth chamber maintaining 60% relative humidity and a 16‐h photoperiod.

### 4.2 Yeast Two‐Hybrid Analysis

The CDS of the target genes were cloned into the pGBKT7 or pGADT7 vectors to generate recombinant plasmids. The recombinant plasmid pGBKT7‐RepA and the cDNA library plasmids of *C. limon* ‘Eureka’ were co‐transformed into Y2H Gold yeast competent cells (Zeng et al. 2023). Subsequently, the transformed yeast cells were plated on selective medium (SD/–Trp–Leu–His–Ade supplemented with 200 ng/mL AbA) and incubated at 28°C for 7 days (Zhao et al. 2017). pGBKT7 and pGADT7 were co‐transformed into Y2H Gold competent cells and plated on SD/–Trp–Leu medium and incubated at 28°C for 3 days. Single colonies were subjected to a ten‐fold gradient dilution using 0.9% NaCl solution and then spotted on the selective medium and incubated at 28°C for 5–7 days (Xie et al. 2024). pGBKT7‐53 and pGADT7‐T co‐transformation served as the positive control; the pGBKT7‐Lam and pGADT7‐T co‐transformation group was considered the negative control (Pant et al. 2020; Wang et al. 2020).

### 4.3 Bimolecular Fluorescence Complementation

RepA and ClMDH were cloned into the pCV‐nYFP and pCV‐cYFP vectors, respectively (Wang et al. 2020). The recombinant plasmids were transformed into *A. tumefaciens* GV3101 and cultured at 28°C with shaking until OD_600_ reached 0.6–0.8 (Zhai et al. 2021). The precipitated agrobacterial cells were resuspended in an inoculation solution (containing 10 mM MES, 10 mM MgCl₂ and 200 μM acetosyringone) to achieve an OD_600_ of 1.0, followed by incubation in the dark for 2 h (Cheng et al. 2021; Zhai et al. 2021). Next, the resuspended agrobacterial cells harbouring different recombinant plasmids were mixed in a 1:1 ratio and injected into *N. benthamiana* leaves (Li et al. 2016). The *Arobacterium*‐infiltrated leaf tissues were observed under a confocal microscope at 40 hpi (Li, Guo, et al. 2022).

### 4.4 Co‐Immunoprecipitation

The CDS of the target gene was cloned into the binary vector, pART27, provided by Professor Xianchao Sun, Southwest University. The pART27‐ClMDH‐3HA‐eGFP and pART27‐RepA‐myc (or pART27‐GUS‐myc as a control) constructs were co‐expressed in *N. benthamiana* leaves via *Agrobacterium* infiltration (Liu et al. 2022). At 48 hpi, total proteins were extracted from the infiltrated *N. benthamiana* leaves using the Solarbio Plant Protein Extraction Kit and incubated with Anti‐GFP Magnetic Beads (BeyoMag) for 3 h at 4°C (Li, Guo, et al. 2022). Next, the beads were washed four times with Tris‐buffered saline (TBS) and then resuspended in 1% SDS‐sample buffer (Cheng et al. 2021). Protein interactions were detected via western blotting using anti‐Myc or anti‐HA antibodies (Han, Zheng, et al. 2023).

### 4.5 Transient Overexpression in *C. limon*

The CDS of the target gene was cloned into the pNmGFPer vector provided by Professor Xiuping Zou, Southwest University. The recombinant plasmid was transformed into *A. tumefaciens* EHA105, and then cultured at 28°C with shaking until OD_600_ reached 0.6–0.8. The precipitated agrobacterial cells were resuspended in the inoculation solution (10 mM MES, 10 mM MgCl_2_ and 200 μM acetosyringone) to achieve an OD_600_ of 1.5, followed by incubation in the dark for 2 h. The resuspended agrobacterial cells harbouring different recombinant plasmids were injected into *C. limon* leaves (Long et al. 2024). Tissue samples were collected at 1, 3, 5 and 7 dpi for RT‐qPCR analysis.

### 4.6 RT‐qPCR

Total RNA was extracted using RNAiso Plus (TaKaRa) and reverse‐transcribed to generate cDNA using All‐In‐One 5 × RT Master Mix (ABM), following the instructions provided by the manufacturers. qPCRs (10 μL) were conducted using 5 μL 2 × SP SYBR qPCR Mix reagent, 0.25 μL of primers with 10 μM primers and 2 μL diluted cDNA (Cheng et al. 2021). The *Actin* and *GADPH* genes were used as housekeeping genes (Pombo et al. 2019; Tian et al. 2025). Relative gene expression levels were calculated using the 2^−ΔΔCt^ method (Tian et al. 2025). Data were processed, analysed, and visualised using GraphPad Prism software (Livak and Schmittgen 2001; Shi, Feng, et al. 2023). The primer sequences are provided in Table S1.

### 4.7 Subcellular Localisation

RepA was fused with the N‐terminus of BFP in the pCV‐BFP vector and ClMDH was fused with the N‐terminus of eGFP in the pART27‐eGFP vector. These constructs were transformed into *A. tumefaciens* GV3101 either individually or together, and infiltrated into *N. benthamiana* as described above. The H2B‐mCherry plasmid was used as a nuclear marker (Zhai et al. 2021). At 40 hpi, fluorescence at the injection site was observed using the Olympus FV3000 confocal microscope. The eGFP fluorescence was excited by 488‐nm laser lines, and the emission was detected at 500–540 nm. The mCherry fluorescence was excited by 561‐nm laser lines, and the emission was detected at 570–620 nm. The autofluorescence from chloroplasts was excited by 640 nm laser lines, and the emission was detected at 650–750 nm (Cheng et al. 2021; Li, et al. 2022).

### 4.8 Bioinformatics Analysis

Homologous gene sequences of *ClMDH* in different citrus varieties were obtained from the CPBD website (Yin et al. 2024). MDH family sequences from various species and the promoter sequences of sweet orange MDHs were acquired from the NCBI website (Cheng et al. 2025). Promoter sequences were submitted to the PlantCARE website for the prediction of cis‐regulatory elements (Zhang et al. 2022). DNAMAN and MEGA software were used for multiple sequence alignment and construction of an unrooted phylogenetic tree, respectively (Wu et al. 2019); TBtools software was used to visualise cis‐regulatory elements (Zhang et al. 2022).

### 4.9 Truncated Mutant Construction

Primers were designed to amplify CDS fragments of varying lengths from the full‐length *ClMDH* CDS. The amplified products were recombined with linearised vectors (pGBKT7 or pART27‐eGFP) using the ClonExpress MultiS One‐Step Cloning Kit (C113) to generate circular plasmids (Cheng et al. 2021).

### 4.10 Western Blot

Total proteins were extracted from leaf tissues using the Solarbio Plant Protein Extraction Kit, and separated through SDS‐PAGE (Mei et al. 2020). Separated proteins were transferred onto polyvinylidene fluoride membranes and blocked with 5% skim milk at room temperature for 2 h. The membranes were washed three times with TBS with Tween 20 (TBST), incubated with primary and secondary antibodies for 1–2 h each, and visualised using a chemiluminescence detection system. ImageJ software was used for quantitative analysis of digital blot images (Zhai et al. 2021).

### 4.11 Diaminobenzidine Staining

The infiltrated leaf samples were collected at 7 dpi, vacuum‐infiltrated with DAB staining solution for 30 min, and then incubated in the dark at room temperature for 12 h (Mei et al. 2020). Chlorophyll was removed from samples by incubating them with 95% ethanol at 85°C using a water bath (Sun et al. 2022; Yang et al. 2024).

### 4.12 VIGS

The SGN‐VIGS website (https://vigs.solgenomics.net/) was utilised to predict a 300 bp specific target for silencing *ClMDH* (Zhang et al. 2023). This fragment was cloned into the TRV2 vector to obtain the recombinant plasmid TRV2:ClMDH (Zhang et al. 2018). The TRV1, TRV2 and TRV2:ClMDH plasmids were separately transformed into the *A. tumefaciens* GV3101. *Agrobacterium* suspensions containing TRV1 and either TRV2 or TRV2:ClMDH were mixed at a 1:1 volume ratio and incubated for 3 h at 24°C. Subsequently, 1‐week‐old lemon seedlings were submerged in the bacterial suspensions for 30 min in a vacuum chamber at 70 kPa (Wang et al. 2019; Long et al. 2024).

### 4.13 Statistical Analyses

Statistical analyses were conducted with GraphPad Prism 9. Data from three independent biological replicates are shown as means ± standard deviation (SD). Differences in gene expression were considered statistically significant at *p < 0.05, **p < 0.01 or ***p < 0.001 based on Student's *t* test.

## Author Contributions

**Yuan Chen:** conceptualization, methodology, software, data curation, investigation, writing – original draft, writing – review and editing, visualization, validation, formal analysis. **Jinfa Zhao:** conceptualization, methodology. **Jiajun Wang:** conceptualization, methodology. **Qi Zhang:** methodology. **Mengji Cao:** supervision, resources. **Yan Zhou:** conceptualization, writing – original draft, writing – review and editing, supervision, resources, funding acquisition, project administration.

## Conflicts of Interest

The authors declare no conflicts of interest.

## Supporting information


**Figure S1:** Subcellular localisation of the truncated mutants ClMDH^Δ1‐41aa^ and ClMDH^Δ42‐412aa^. H2B‐mCherry is a nuclear marker with red fluorescence. Scale bar, 20 μm. Each experiment was repeated three times, and each repeat included three biological replicates.

**Figure S2:** Homology analysis of ClMDH family and cis‐regulatory element analysis. (a) The phylogenetic tree constructed by aligning the protein sequences of ClMDH with 43 homologous MDH sequences from *Citrus sinensis* , *Arabidopsis thaliana* , *Triticum aestivum* , *Oryza sativa* , *Zea mays* and other citrus varieties. (b) Left: Unrooted phylogenetic tree constructed based on seven *C. sinensis* MDH proteins, right: schematic diagram of cis‐regulatory elements in the upstream 2000 bp promoter regions of these MDHs. Displayed elements include MYB, MYC, STRE, ABRE, LTR, G‐box, Box 4, I‐box, P‐box, GARE‐motif, TC‐rich repeats, CGTCA‐motif, TGACG‐motif and GT1‐motif.

**Figure S3:** Multiple sequences alignment results of plNAD‐MDHs. (a) Multiple sequences alignment of ClMDH with plNAD‐MDHs from the *Citrus*. (b) Multiple sequence alignment of ClMDH with plNAD‐MDHs from four species: *Citrus limon* (ClMDH), *Arabidopsis thaliana* (AT3G47520), *Oryza sativa* (NP 001390129.1, XP 066159736.1, NP 001396023.1) and *Zea mays* (NP 001307728.1).

**Figure S4:** Western blot analysis of RepA‐mediated impairment on ClMDH‐induced PCC assay.

**Table S1:** RT‐qPCR primer sequences.

## Data Availability

The data that support the findings of this study are available from the corresponding author upon reasonable request.
