# Supplementary figures and images for: RepA Protein of Citrus Chlorotic Dwarf‐Associated Virus Impairs Perinuclear Chloroplast Clustering Induced by Lemon Chloroplast Malate Dehydrogenase
Source: Mol Plant Pathol. 2025 Aug 7;26(8):e70133. doi: 10.1111/mpp.70133 (PMC12330936; doi:10.1111/mpp.70133)

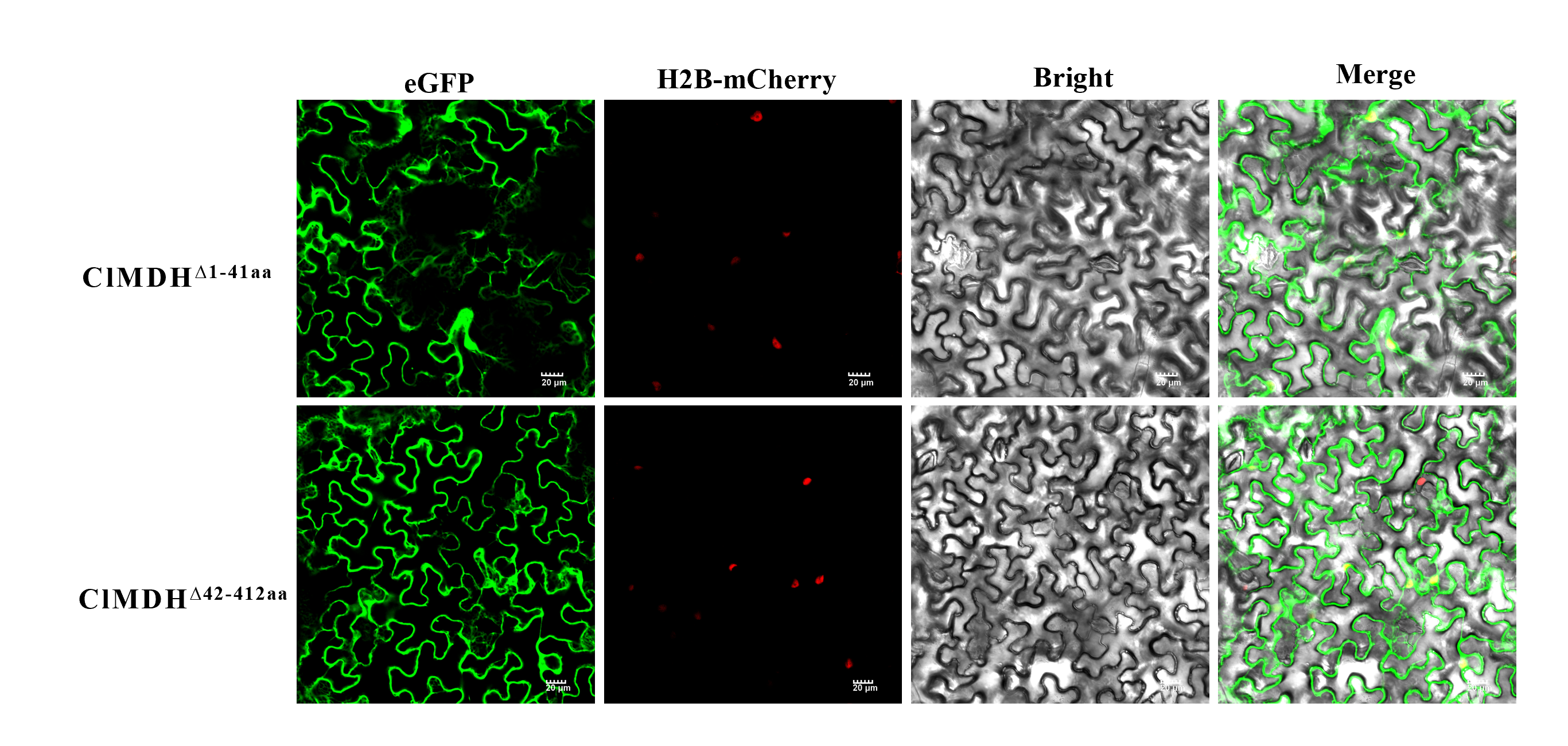

Supplement: Supplementary file 1 — Figure S1: Subcellular localisation of the truncated mutants ClMDHΔ1‐41aa and ClMDHΔ42‐412aa. H2B‐mCherry is a nuclear marker with red fluorescence. Scale bar, 20 μm. Each experiment was repeated three times, and each repeat included three biological replicates. [file MPP-26-e70133-s004.tif]

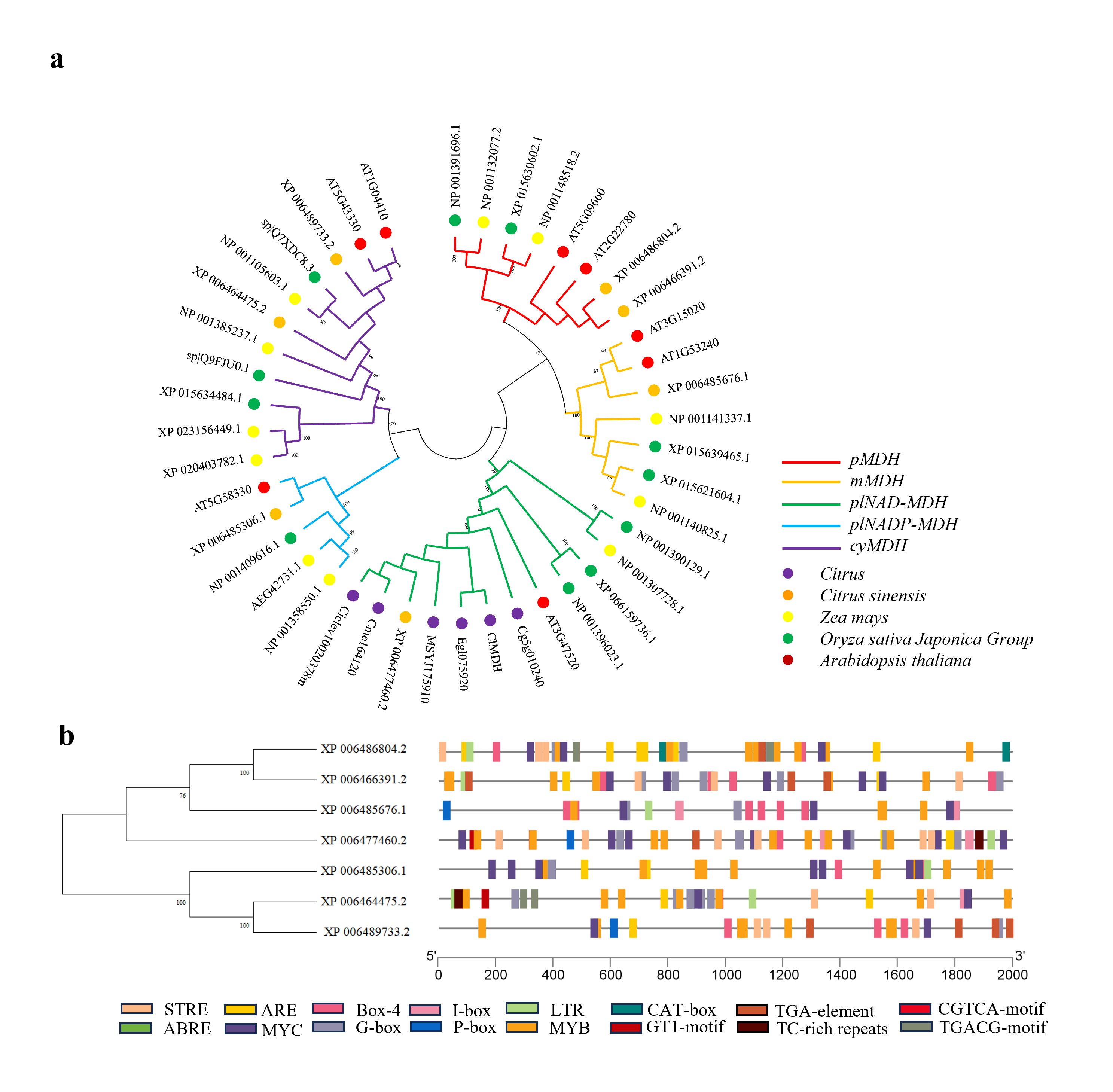

Supplement: Supplementary file 2 — Figure S2: Homology analysis of ClMDH family and cis‐regulatory element analysis. (a) The phylogenetic tree constructed by aligning the protein sequences of ClMDH with 43 homologous MDH sequences from Citrus sinensis , Arabidopsis thaliana , Triticum aestivum , Oryza sativa , Zea mays and other citrus varieties. (b) Left: Unrooted phylogenetic tree constructed based on seven C. sinensis MDH proteins, right: schematic diagram of cis‐regulatory elements in the upstream 2000 bp promoter regions of these MDHs. Displayed elements include MYB, MYC, STRE, ABRE, LTR, G‐box, Box 4, I‐box, P‐box, GARE‐motif, TC‐rich repeats, CGTCA‐motif, TGACG‐motif and GT1‐motif. [file MPP-26-e70133-s002.tif]

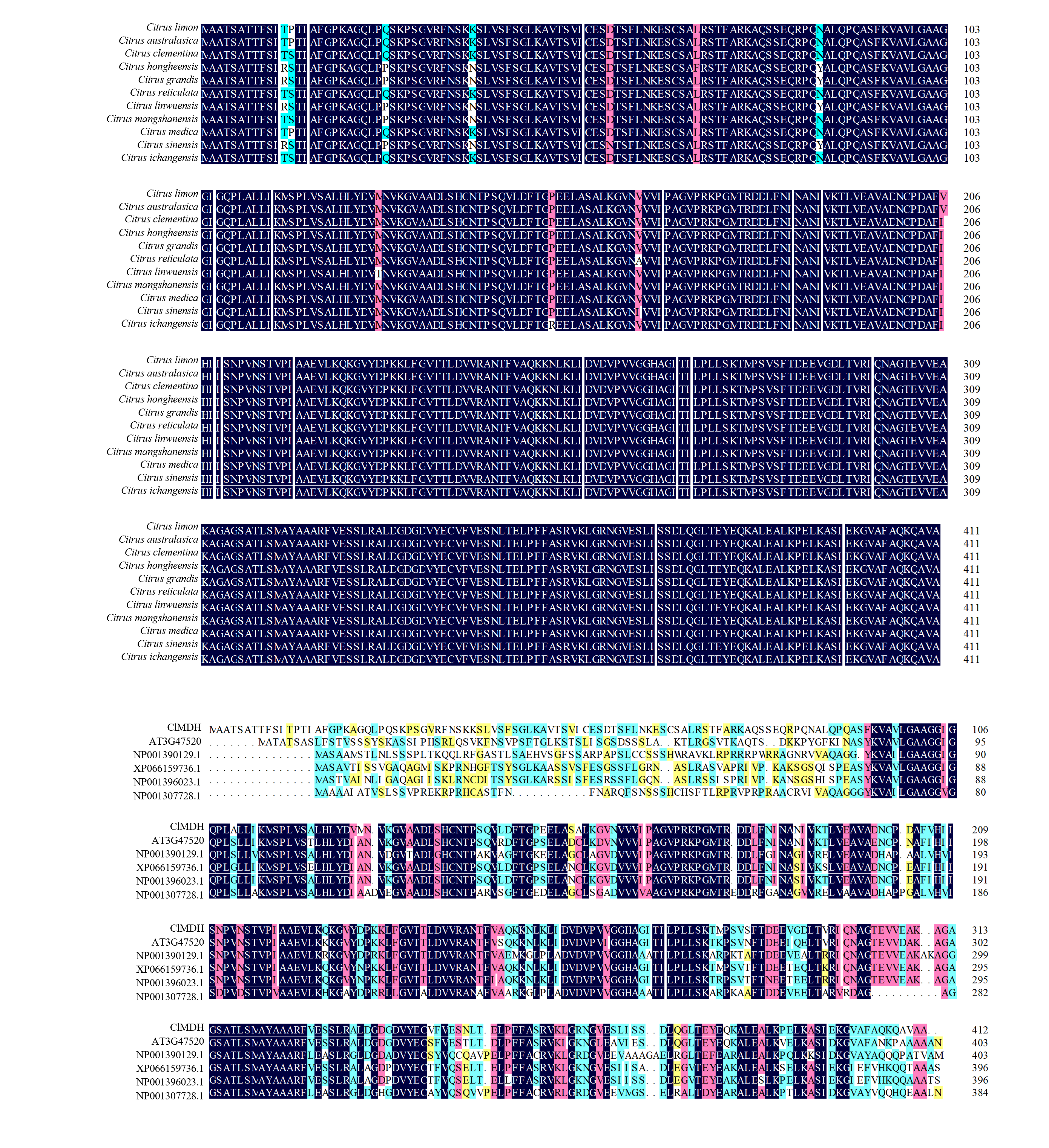

Supplement: Supplementary file 3 — Figure S3: Multiple sequences alignment results of plNAD‐MDHs. (a) Multiple sequences alignment of ClMDH with plNAD‐MDHs from the Citrus. (b) Multiple sequence alignment of ClMDH with plNAD‐MDHs from four species: Citrus limon (ClMDH), Arabidopsis thaliana (AT3G47520), Oryza sativa (NP 001390129.1, XP 066159736.1, NP 001396023.1) and Zea mays (NP 001307728.1). [file MPP-26-e70133-s001.tif]

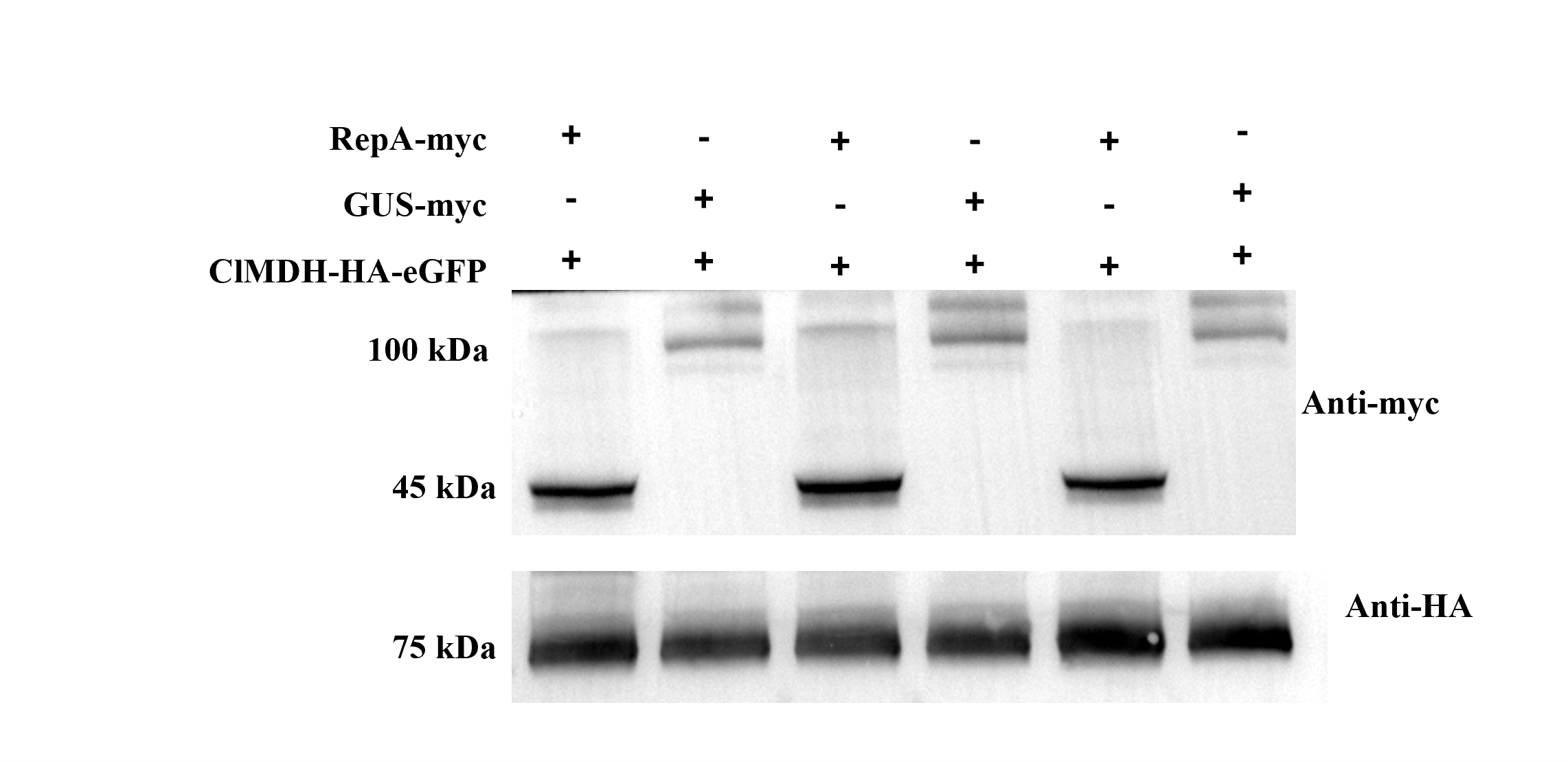

Supplement: Supplementary file 4 — Figure S4: Western blot analysis of RepA‐mediated impairment on ClMDH‐induced PCC assay. [file MPP-26-e70133-s005.tif]
